# Effects of Preoperative Exercise Interventions in Patients Undergoing Metabolic and Bariatric Surgery: A Systematic Review and Meta-Analysis

**DOI:** 10.3390/jcm14176170

**Published:** 2025-09-01

**Authors:** Daniel Simancas-Racines, Juan Marcos Parise-Vasco, Jaime Angamarca-Iguago, Ashley Carolina Cuzco-Macias, Carlos Soria, Salvatore Tramontano, Gianluca Rossetti, Francesco Cobellis, Luigi Cobellis, Vincenzo Pilone, Luigi Barrea, Evelyn Frias-Toral, Claudia Reytor-González, Luigi Schiavo

**Affiliations:** 1 Universidad UTE, Facultad de Ciencias de la Salud Eugenio Espejo, Centro de Investigación en Salud Pública y Epidemiología Clínica (CISPEC), Quito 170527, Ecuador; juan.parise@ute.edu.ec (J.M.P.-V.); jaime.angamarca@ute.edu.ec (J.A.-I.); claudia.reytor@ute.edu.ec (C.R.-G.); 2 Servicio de Cardiología, Hospital General de Agudos Dr. Juan A. Fernández, Buenos Aires C1425AGP, Argentina; ashley.cusco@ute.edu.ec; 3 Universidad UTE, Posgrados de Ciencias de la Salud, Maestría de Epidemiología Con Mención en Investigación Clínica Aplicada, Quito 170527, Ecuador; 4 Hospital de Lima Este Vitarte, Ctra. Central KM 7.5, Ate 15491, Peru; carlos.soria.343@gmail.com; 5 General and Emergency Surgical Unit, Fucito Hospital, San Giovanni di Dio e Ruggi d’Aragona University Hospital, 84131 Salerno, Italy; salvytra@libero.it; 6 General and Bariatric Surgery Unit, Abano Terme Policlinic, 35031 Padova, Italy; gianlucarossetti@yahoo.it; 7 Unit of General Surgery, Casa Di Cura “Prof. Dott. Luigi Cobellis”, 84078 Vallo della Lucania, Italy; francesco.cobellis@unina.it (F.C.); luicobellis@yahoo.it (L.C.); 8 Public Health Department, University of Naples Federico II, 80131 Naples, Italy; vicenzo.pilone@unina.it; 9 Dipartimento di Psicologia e Scienze della Salute, Università Telematica Pegaso, Centro Direzionale, Via Porzio, Isola F2, 80143 Napoli, Italy; luigi.barrea@unipegaso.it; 10 Escuela de Medicina, Universidad Espíritu Santo, Samborondón 0901952, Ecuador; evelynft@gmail.com; 11 Division of Research, Texas State University, 601 University Dr., San Marcos, TX 78666, USA; 12 Department of Medicine, Surgery and Dentistry “Scuola Medica Salernitana”, University of Salerno, 84081 Baronissi, Italy; lschiavo@unisa.it; 13 National Biodiversity Future Center (NBFC), 90133 Palermo, Italy

**Keywords:** bariatric surgery, preoperative exercise, functional capacity, cardiorespiratory fitness, meta-analysis

## Abstract

**Background**: Obesity affects over one billion people globally. Bariatric surgery is the most effective long-term intervention for severe obesity. However, postoperative outcomes can vary considerably, with such factors as baseline fitness and cardiorespiratory reserve influencing surgical outcomes. This systematic review aimed to evaluate the effects of preoperative exercise or physical activity, compared to standard care or no intervention, on preoperative fitness parameters and perioperative surgical outcomes in adults with obesity undergoing metabolic and bariatric surgery. **Methods**: A systematic review was conducted in accordance with the recommendations of the *Cochrane Handbook* and the PRISMA guidelines. Randomized controlled trials, non-randomized controlled trials, and cohort studies with control groups evaluating preoperative exercise interventions were included. Two independent reviewers conducted study selection, data extraction, and risk of bias assessment using Cochrane tools. Meta-analyses were performed using random effects models, with standardized mean differences calculated for continuous outcomes. Evidence certainty was assessed using the GRADE approach. **Results**: A total of 15 studies, including 1378 participants, were identified for qualitative synthesis, with 12 contributing data for quantitative meta-analysis. Preoperative exercise interventions significantly improved six-minute walk test distance (SMD 2.01; 95% CI: 0.51 to 3.50; *p* = 0.009) and VO_2_ peak (SMD 1.02; 95% CI: 0.52 to 1.51; *p* < 0.0001). BMI reduction was significant (SMD −0.96; 95% CI: −1.75 to −0.16; *p* = 0.02), while weight change was not statistically significant (SMD −0.81; 95% CI: −1.72 to 0.09; *p* = 0.08). One study reported a reduction in hospital length of stay of 0.64 days (95% CI: −0.86 to −0.42; *p* < 0.00001). Evidence certainty was rated as very low to low across all outcomes. **Conclusions**: Preoperative exercise interventions have been shown to significantly improve cardiorespiratory fitness in bariatric surgery candidates, with large effect sizes for functional capacity measures. Despite the low certainty of the evidence, these findings suggest that supervised exercise programs should be incorporated into the preoperative care of bariatric surgery patients.

## 1. Introduction

Obesity has emerged as one of the most pressing global health challenges of the 21st century, with its prevalence increasing at an alarming rate across all regions of the world [1]. According to the World Health Organization, more than one billion people were living with obesity in 2022, representing approximately 16% of the global adult population [2]. While historically more prevalent in high-income settings, recent trends indicate a rapid rise in obesity rates across low- and middle-income countries, where it increasingly coexists with undernutrition—a phenomenon known as the double burden of malnutrition [3,4]. This epidemiological shift reflects profound changes in global food systems, urbanization, sedentary lifestyles, and socioeconomic inequities [5,6,7,8]. Beyond its growing prevalence, obesity is strongly linked to a wide range of chronic conditions, including type 2 diabetes [9], cardiovascular disease [10], certain types of cancer [11,12], and musculoskeletal disorders [13], all of which contribute significantly to global morbidity, disability, and premature mortality [14].

For individuals with severe obesity, bariatric surgery (BS) stands as the most effective long-term intervention, facilitating substantial weight loss and improvement in obesity-related comorbidities [15]. However, postoperative outcomes exhibit considerable variability [16,17]. Factors, such as baseline fitness, systemic inflammation, and cardiorespiratory reserve, can influence surgical and functional outcomes. Given that patients with obesity often present with poor physical conditioning and reduced aerobic capacity [18], there is growing interest in identifying modifiable factors that could improve perioperative outcomes [19]. Emerging evidence also suggests that targeted preoperative interventions—whether nutritional or physical—may play a role in mitigating perioperative risk and optimizing recovery trajectories [20,21,22,23,24,25,26,27].

Preoperative exercise interventions—or prehabilitation—have emerged as a promising strategy to enhance physiological resilience before major surgery [28,29,30]. These interventions typically include aerobic training, resistance exercises, or multimodal programs tailored to the individual’s functional status [31]. Among patients undergoing BS, preoperative exercise may confer several benefits. A systematic review by Durey et al. demonstrated that preoperative exercise interventions led to significant improvements in cardiorespiratory fitness, as measured by maximum rate of oxygen consumption (VO_2_max), both before surgery and at follow-up [32]. Similarly, Ghannadi et al. reported that engaging in pre-bariatric metabolic surgery exercise training might improve anthropometric parameters and cardiopulmonary fitness [33]. Consistently, Bellchica et al. found that exercise training—particularly after bariatric surgery—significantly increased VO_2_max and muscle strength, further supporting the physiological benefits of structured physical activity in this population [34]. However, these studies also highlight the heterogeneity in exercise protocols and outcomes assessed, underscoring the need for standardized approaches.

Despite these promising findings, the existing literature on preoperative exercise in BS patients is heterogeneous, with variations in study designs, exercise protocols, and measured outcomes. This heterogeneity poses challenges in drawing definitive conclusions and developing standardized guidelines.

Given the rising demand for bariatric procedures and the need to optimize surgical safety and effectiveness, a comprehensive synthesis of the available evidence is warranted. Therefore, this systematic review aims to evaluate the effects of preoperative exercise or physical activity, compared to standard care or no intervention, on both preoperative conditioning outcomes and perioperative surgical outcomes in adults with obesity undergoing BS, applying the GRADE approach to assess evidence certainty.

## 2. Methods

A systematic review was conducted to assess the effectiveness of preoperative exercise or physical activity interventions on surgical outcomes in patients undergoing metabolic and bariatric surgery. The methodology followed the recommendations of the *Cochrane Handbook for Systematic Reviews of Interventions* [35], and reporting followed the Preferred Reporting Items for Systematic Reviews and Meta-Analysis (PRISMA) guidelines [36]. The review protocol was prospectively registered in PROSPERO (CRD42024622203). The research question was structured according to the PICO framework: Population (P): Adult patients with obesity undergoing metabolic and bariatric surgery; Intervention (I): Preoperative exercise or physical activity; Comparison (C): Standard care, any other preoperative intervention, or no intervention; Outcome (O): Operative time (minutes), length of hospital stay (days), postoperative complications, weight changes (kg), BMI changes, cardiorespiratory fitness (VO_2_max, 6-min walk test), and metabolic parameters (changes in insulin sensitivity, lipid profile).

### 2.1. Search Strategy

Six bibliographic databases were systematically queried, namely MEDLINE via PubMed, the Cochrane trials registry, Latin American databases (LILACS, BVS), Scopus, and Epistemonikos. Publications in English and Spanish through November 2024 were eligible. Search algorithms integrated concepts of weight-reduction surgery, metabolic procedures, exercise training, and pre-surgical timing. Detailed search strategies are presented in Table S1.

Furthermore, we examined clinical trial registries, such as ClinicalTrials.gov and the World Health Organization International Clinical Trials Registry Platform (WHO ICTRP). Gray literature was located through a search on Google Scholar. Reference lists of included research and pertinent systematic reviews were manually reviewed to identify additional eligible studies.

### 2.2. Eligibility Criteria

We included randomized controlled trials, non-randomized controlled clinical trials, and cohort studies with control groups that evaluated preoperative exercise or physical activity interventions in patients undergoing metabolic and bariatric surgery. Studies had to include adult patients and report at least one of the following outcomes: operative time, length of hospital stay, postoperative complications, changes in weight or BMI, cardiorespiratory fitness parameters, or metabolic parameters. We excluded systematic reviews, case reports, letters to the editor, studies without a comparison group, studies that did not specifically analyze preoperative exercise or physical activity interventions, and studies that focused exclusively on other preoperative interventions, such as dietary treatments, pharmacological treatments, or intragastric balloons.

### 2.3. Study Selection

The study screening employed a dual-reviewer approach (JMPV and ACCM), utilizing the systematic management software Rayyan (Qatar Computing Research Institute, Doha, Qatar; https://rayyan.qcri.org, accessed on 24 December 2024). A hierarchical evaluation strategy was implemented: initial title and abstract assessment identified potentially relevant citations, followed by a thorough examination of the full text to verify adherence to the eligibility criteria. Any discrepancies were resolved through discussion, with a third evaluator (DSR) providing arbitration if necessary.

### 2.4. Data Extraction

Data extraction was performed utilizing a standardized matrix by three reviewers (JMPV, ACCM, and JAI), who collaborated to achieve consensus on the extracted data elements. The following information was extracted from each study: title, study characteristics, patient demographics, details of exercise or physical activity interventions, including type, intensity, duration, frequency, surgical procedure performed, funding sources and conflicts of interest declarations, and outcome measures, such as operative time, hospital stay, complications, changes in weight and BMI, cardiorespiratory parameters, and metabolic parameters. Any discrepancies that arose during the data extraction process were resolved through consensus with a fourth reviewer (DSR).

### 2.5. Risk Bias Assessment of Included Studies

Two evaluators (JMPV and ACCM) independently appraised methodological quality, applying the Cochrane Risk of Bias tool for randomized controlled trials, and the ROBINS-I tool for non-randomized trials [37]. Disagreements in the quality assessment were resolved by consensus or arbitration by a third reviewer (DSR).

### 2.6. Data Synthesis and Statistical Analysis

A qualitative synthesis of study characteristics, interventions, and outcomes was conducted. For quantitative meta-analysis, trials comparing preoperative exercise or physical activity interventions with standard care or any other comparator were pooled, regardless of the specific duration and intensity of the exercise intervention. This approach was chosen due to the heterogeneity in the definitions and protocols of exercise interventions in the included studies.

Model selection (fixed versus random effects) depended on an evaluation of heterogeneity, both clinical and statistical. Due to the significant variation among the eligible trials, we primarily employed random effects modeling. Statistical heterogeneity was evaluated using the I^2^ statistic, with values exceeding 50% indicating substantial heterogeneity that warranted random-effects estimates. When I^2^ remained at or below 50%, sensitivity analyses were conducted to compare the results of both models [35].

Effect measures were selected based on the nature and quality of available data for each outcome. For continuous outcomes where standard deviations were frequently imputed or calculated indirectly using methods, such as converting confidence intervals, *p*-values, or standard errors, standardized mean differences (SMD) with 95% confidence intervals were used as the primary effect measure. This approach was adopted because indirect estimation of standard deviations introduces inherent uncertainty and potential variation in the precision of estimates across studies, which standardized mean difference effectively addresses by normalizing effect sizes relative to the pooled standard deviation. For length of hospital stay, mean differences (MD) with 95% confidence intervals were used due to the availability of direct standard deviation estimates and the clinical interpretability of results in days. Standardized mean differences were interpreted using established Cohen’s conventions, where values of 0.2, 0.5, and 0.8 represent small, medium, and large effect sizes, respectively [35]. The data were analyzed using the Review Manager (RevMan) v5.3 software. The certainty of the evidence for each outcome was then assessed using the Grading of Recommendations Assessment, Development, and Evaluation (GRADE) approach with GRADEpro GDT software (McMaster University, Hamilton, Canada) [38].

Publication bias was planned to be assessed using funnel plots and Egger’s test when ten or more studies could be pooled for an outcome. However, as fewer than ten trials were available for the meta-analysis of each outcome, formal assessment of publication bias could not be conducted.

## 3. Results

**Figure 1:**
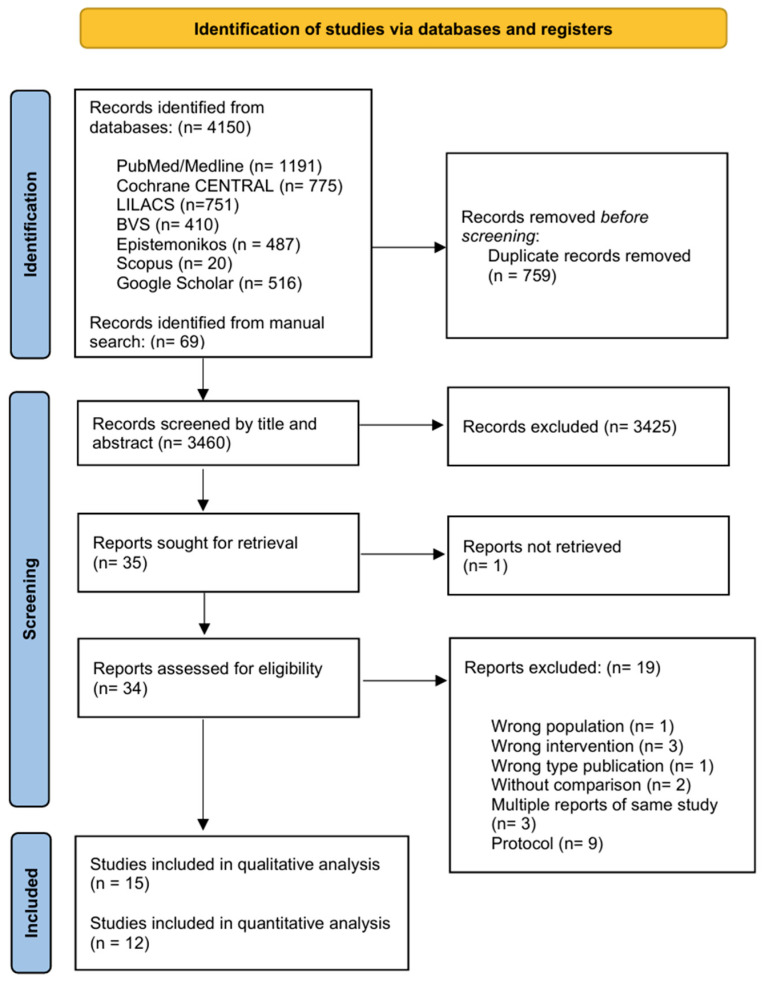
PRISMA flowchart of systematic review selection process and included studies.

A total of 4219 records were identified through the systematic search, including 4150 through electronic database searches and 69 through manual searches. After removing 759 duplicates, the remaining 3460 records were screened by title and abstract, resulting in the exclusion of a further 3425 records. Following the initial screening, 35 full-text articles were assessed for eligibility. One article could not be retrieved, leaving thirty-four reports to be reviewed in full. Of these, 19 were excluded for the following reasons: wrong population (*n* = 1), wrong intervention (*n* = 3), wrong type of publication (*n* = 1), no comparison group (*n* = 2), multiple reports of the same study (*n* = 3), and protocol papers (*n* = 9) [39,40,41,42,43,44,45,46,47,48,49,50,51,52,53,54,55,56] (Table S2). The final analysis included fifteen studies for qualitative synthesis [28,29,57,58,59,60,61,62,63,64,65,66,67,68,69], and twelve studies with data for quantitative meta-analysis [28,29,57,58,61,62,63,64,65,66,67,68]. This process is illustrated in the PRISMA flow diagram (Figure 1). Table 1 summarizes the key characteristics of all included studies.

### 3.1. Characteristics of Studies

#### 3.1.1. Geographic Distribution and Settings

The included studies were conducted across multiple regions, with the majority originating from North America, including studies from the United States [28,59,60,62,64,65,69] and Canada [29,58]. European countries contributed with studies from Spain [66,67,68], Turkey [57], and the Netherlands [61]. One study was conducted in Brazil [63]. Most interventions were implemented in hospital-based or university-affiliated facilities, with some incorporating community-based or home-based components.

#### 3.1.2. Participant Characteristics

The 18 studies included a total of 1378 participants ranging from 7 to 884 participants per study [65,69]. The mean age across studies ranged from 37.25 to 50.1 years [58,65], with the majority including middle-aged adults. Female participants comprised the majority across most studies, with percentages ranging from 78% to 100% in individual studies [58,63,69].

Baseline body mass index (BMI) values varied across studies, with mean BMI ranging from 38.78 to 51.3 kg/m^2^ [67,69]. The majority of participants were classified as having class III obesity (BMI ≥ 40 kg/m^2^), though some studies included participants with class II obesity (BMI 35–39.9 kg/m^2^) when accompanied by significant comorbidities [66].

The most common surgical procedure was the Roux-en-Y gastric bypass (RYGB). This procedure was performed in four studies [28,62,65,69], and represented the majority of procedures in the remaining studies. Sleeve gastrectomy was performed in three studies, either as the sole intervention or in combination with RYGB [28,29,60]. Three studies included laparoscopic adjustable gastric banding (LAGB), with one evaluating it as the primary procedure [58,60,64]. One study included biliopancreatic diversion with duodenal switch in a small proportion of cases [60]. Eight studies did not specify the type of bariatric procedure performed, referring to it only as bariatric surgery or metabolic surgery [29,57,59,61,63,66,67,68].

#### 3.1.3. Exercise Intervention Characteristics

##### Exercise Modality and Design

The exercise interventions varied considerably across studies. Combined aerobic and resistance training was the most common approach, implemented in eight studies [29,58,60,61,62,66,67,68]. Four studies focused exclusively on aerobic exercise interventions [28,59,63,65]. Two studies implemented specialized approaches: one focused on core stabilization exercises [57] and another focused on aquatic exercise [65]. One study investigated a medically supervised weight management program that included physical activity counseling [64].

##### Duration and Frequency

Intervention durations ranged from 30 days [28,59] to 6 months [67,69], with the most common duration being 12 weeks [29,57,60,66,68]. Weekly frequency varied from 2–4 sessions per week, with most studies implementing 2–3 sessions weekly. For example, Arman et al. [57] prescribed two sessions per week, while Hardy et al. [29] implemented three sessions per week. Session duration typically ranged from 30 to 80 min, with Arman et al. [57] using 30 to 45 min sessions and Baillot et al. [58] implementing 80 min sessions, and with many studies employing progressive increases in duration throughout the intervention period.

##### Supervision and Setting

Supervision levels varied across studies. Nine studies implemented fully supervised interventions with qualified exercise professionals [29,57,58,60,63,65,66,67,68], while two studies used a partially supervised approach [59,61]. Four studies did not clearly specify the level of supervision provided [28,62,64,69]. Most supervised interventions took place in hospital-based fitness facilities [29,65], university exercise laboratories [67,68], or clinical settings [60], while some studies incorporated home-based components [59,62].

##### Exercise Intensity

Exercise intensity prescription varied considerably across studies. Four studies used heart rate-based intensity prescription, typically targeting 65–85% of maximum heart rate or heart rate reserve [28,29,59,62]. Four studies employed perceived exertion scales, with targets typically ranging from 13–15 on the Borg scale [57,58,63,65]. Three studies utilized specific training zones, such as the intensity of maximum fat oxidation (Fatmax) or ventilatory thresholds [66,67,68]. Several studies did not specify intensity parameters or used qualitative descriptors, such as “moderate” or “low” intensity [60,61,64,69].

#### 3.1.4. Control Group Characteristics

Control groups received standard preoperative care in all studies, which typically included routine medical, nutritional, and psychological consultations. Several studies mandated specific preoperative dietary interventions, such as very low-calorie liquid diets 2–3 weeks before surgery [29,58,69]. Some control groups received basic physical activity counseling or educational materials without structured exercise programming [60,64]. García-Delgado et al. [61] provided cognitive behavioral group sessions as part of standard care, while Still et al. [69] implemented a comprehensive multidisciplinary program including behavioral modules and activity encouragement.

#### 3.1.5. Funding Sources and Conflicts of Interest

Funding information was extracted from all 15 included studies. Ten studies (67%) reported specific funding sources: governmental or institutional grants were most common, including the Canadian Institutes of Health Research [43], NIH grants [58], university internal awards [32], and national/regional research funds [44,46,48,52]. Three studies explicitly stated receiving no external funding [33,51] or no financial support [42]. Funding information was not reported in two studies [47,50,53].

Regarding conflicts of interest, twelve studies (80%) explicitly declared no conflicts of interest [32,33,42,43,45,46,48,50,51,52,53]. One study reported specific disclosures, with two authors on a medical advisory board unrelated to the study intervention [44]. One study reported a co-author receiving research support from a company unrelated to the exercise intervention [58]. Conflict of interest statements were not found in two studies [47,49]. Notably, no studies reported funding from exercise equipment manufacturers or bariatric surgery device companies, reducing concerns about industry bias in the exercise intervention outcomes. Complete funding details are provided in Supplementary Table S3.

### 3.2. Qualitative Synthesis of Key Outcomes

This qualitative synthesis is based on data extracted from reports by Arman et al. [57], Baillot et al. [58], Bond et al. [59], Creel et al. [60], Funderburk et al. [65], García-Delgado et al. [61], Gilbertson et al. [28], Hardy et al. [29], Li et al. [62], Marc-Hernández et al. [66], Marcon et al. [63], Parikh et al. [64], Pico-Sirvent et al. [67,68], and Still et al. [69]. These studies investigated various preoperative exercise interventions in adult candidates for metabolic and bariatric surgery. It should be noted that several reports represent pilot studies or poster presentations [28,62,65,66], and data for some studies were limited or fragmentary in the provided extraction [64,69]. The study by Still et al. [69] appeared observational and focused on preoperative weight loss associations rather than a structured exercise intervention.

#### 3.2.1. Intervention Characteristics

Preoperative interventions varied considerably across the studies. Durations ranged from 4 weeks [61,67] and 30 days [28] up to 12 weeks [58,59]. Session frequency was typically 2–3 times per week. Intervention types included core stabilization exercises [57], combined aerobic and resistance training [29,58,67], primarily aerobic training [28,59], multimodal programs incorporating resistance training, inspiratory muscle training, and nutritional support [61,66], and behavioral counseling approaches focusing on increasing physical activity [59,60]. Supervision levels also varied, with many programs being supervised by physiotherapists or exercise specialists [29,57,58,59,61], while others involved non-supervised or collaborative goal-setting approaches [60,62]. Control groups typically received standard preoperative care, which often included general physical activity counseling but no structured exercise program.

#### 3.2.2. Surgical Parameters

Data on surgical parameters were sparsely reported in the extracted summaries. Most studies did not analyze operating time or length of hospital stay [28,29,57,58,59,60,62,63,65,67]. However, a 4-week multimodal prehabilitation program (resistance training, inspiratory muscle training, protein supplementation) resulted in significantly fewer postoperative complications (3/30 events) compared to standard care (9/30 events, *p* = 0.02) in patients undergoing laparoscopic sleeve gastrectomy [61,66].

#### 3.2.3. Body Composition

Preoperative exercise interventions generally resulted in minimal or non-significant changes in body weight and BMI prior to surgery. Studies implementing core stabilization [57], combined aerobic/resistance training [58], or behavioral physical activity interventions [59] reported no statistically significant changes in weight or BMI over 8–12 weeks. Similarly, fat mass changes were often non-significant [57]. However, some interventions demonstrated modest but statistically significant reductions. A 4-week multimodal program led to a mean weight loss of 2.3 kg (*p* = 0.001) and a 1.1% reduction in fat mass (*p* = 0.001) [61], as shown by Marc-Hernández et al. [66]. Studies by Marcon et al. [63] and Pico-Sirvent et al. [67] also reported significant weight/BMI reductions (*p* < 0.05), although quantitative data were limited in the extraction. One study found no significant difference in % excess weight loss at 10 weeks post-surgery between supervised, non-supervised, and control groups, despite trends favoring the exercise groups [62].

#### 3.2.4. Functional Capacity

Improvements in functional capacity were a consistent finding across multiple studies employing diverse interventions. Significant increases in 6-min walk test (6MWT) distance were observed following core stabilization (mean change +146 m, *p* = 0.005; [57]), combined aerobic/resistance training (+26 m, *p* = 0.02 in completers; [58]; +48 m, *p* = 0.001; [61,66]; significant improvement, *p* < 0.05; [63]; significant improvement, *p* < 0.05; [67]). Effect sizes for 6MWT improvements were often moderate to large (e.g., Cohen’s d = −0.78 [57]; d = −0.45 [61]).

Cardiorespiratory fitness, assessed via VO_2_max or maximal exercise testing, also improved significantly in intervention groups compared to controls in several studies (Baillot et al. [58]: +1.0 mL/kg/min, *p* = 0.02 in completers; Hardy et al. [29]: *p* < 0.05; Marcon et al. [63]: *p* < 0.05). A collaborative, non-prescriptive intervention also improved METs achieved during submaximal testing (+1.0 METs, *p* = 0.02 [60]). One pilot study showed a trend towards improved VO_2_max preoperatively in a supervised group (+2.5 mL/kg/min, *p* = 0.15), but this was not statistically significant [62].

Muscle strength gains were reported following interventions incorporating resistance training. Handgrip strength increased significantly (+3.5 kg, *p* = 0.001, Cohen’s d = −0.62) [61,66], and leg press peak torque increased by 15% (*p* < 0.05) [67].

Objectively measured physical activity levels increased significantly in response to behavioral interventions. Bond et al. [59] found significant increases in daily steps (+1600 steps/day, *p* = 0.001) and moderate-to-vigorous physical activity (MVPA) (+16.6 min/day, *p* = 0.001, Cohen’s d = −0.40) compared to standard care over 6–12 weeks. These improvements in physical activity were partially maintained at 6 months post-surgery in the intervention group [59]. Self-reported physical activity also increased [57,58], alongside reductions in sitting time [57,59].

#### 3.2.5. Metabolic Parameters

Data on metabolic parameters were limited. Two reports by Gilbertson et al. [28] indicated that a 30-day preoperative aerobic exercise program improved markers of cardiometabolic health, including arterial stiffness, C-reactive protein (CRP), and cytokeratin 18 (all *p* < 0.05), compared to standard care. Pico-Sirvent et al. [68] reported that an unspecified intervention led to a significant decrease in resting carbohydrate oxidation (−10%, *p* < 0.05) and an increase in resting fat oxidation (+15%, *p* < 0.05), suggesting favorable shifts in substrate utilization, although resting metabolic rate and fat-free mass were unchanged. Many studies did not report on insulin sensitivity or lipid profiles [29,57,58,59,61,62,63,65,67].

#### 3.2.6. Other Outcomes

Improvements in patient-reported outcomes were noted. Arman et al. [57] found significant reductions in fatigue (FSS score change −2.70, *p* = 0.005) and improvements in obesity-specific quality of life (OSQOL score change −15.55, *p* = 0.005) following a core stabilization program. Baillot et al. [58] reported improved social interaction scores (*p* = 0.02) on the Laval questionnaire after a combined exercise program. A pilot study by Funderburk et al. [65] observed a significant reduction in depressive symptoms (BDI score change −7.75, *p* = 0.005) after a 6-week exercise program, although anxiety and SF-36 physical functioning scores did not change significantly. Arman et al. [57] also reported significant improvements in postural stability (*p* = 0.018) and fall risk (*p* = 0.012).

**Table 1:** Characteristics of the included studies.

Study (Author, Year)	Study Design	Population (N; BMI kg/m^2^; Age yrs; % Female)	Intervention (Type; Duration; Frequency; Supervision)	Control Group	Key Outcomes Measured	Follow-Up Duration
Gilbertson et al. (2020) [28]	Controlled pilot study	N = 14 completed (SC = 7, EX + SC = 7); BMI: NR; age: ~39–46 (SC 39.0 ± 5.3, EX + SC 45.6 ± 4.8); female: 13/14 (92.8%) (SC 6/7, EX + SC 7/7)	Aerobic exercise + standard care + Low-calorie diet; 30 days; frequency NR; supervised (inferred from authors)	Standard care (includes diet)	Metabolic health (insulin sensitivity, glucose tAUC), adipokines (mentioned), cardiometabolic health (arterial stiffness surrogate (AIx@75), CRP, CK18), body composition (weight, FFM, and WC), and weight-related quality of life	30 days pre-op
Hardy et al. (2022) [29]	Pilot randomized controlled trial	N = 54 enrolled, N = 52 in tables; BMI: ~45.75 (Cont 45.2, Int 46.3); age: NR; female: Cont 63.0%, Int 88.0%	Supervised exercise program (aerobic + resistance); 12 weeks; 3x/week (inferred from citation); supervised	Standard multidisciplinary preoperative care	Functional capacity (6MWT primary, GXT, VO_2_max), strength (chair stand, handgrip), flexibility (sit and reach), quality of life (Laval questionnaire: activity, symptoms, hygiene, emotions, social, and sex), anthropometric (Wt, BMI, neck, waist, and hip), and balance	12 weeks pre-op
Arman et al. (2021) [57]	Randomized controlled trial (RCT)	N = 21 analyzed (EG = 10, CG = 11); BMI: NR; age: NR; female: NR	Core stabilization exercise program + physical activity counseling (CSEP); 8 weeks; 2x/week; supervised	Physical activity counseling only	Body composition (using Tanita BC420MA), balance (PST, FRT, and BCT with Biodex Balance System), fatigue (Fatigue Severity Scale), and functional capacity (6MWT)	8 weeks pre-op
Baillot et al. (2016) [58]	Randomized controlled trial (RCT)	N = 30 randomized; N = 21 completers; BMI: 47.5 ± 8.1, median ~45; age: 43.2 ± 9.2; female: NR in (percentages per group NR)	Pre-surgical exercise training (PreSET) (endurance and strength training) + interdisciplinary lifestyle management; 12 weeks; 3x/week; supervised	Usual care (includes counseling)	Physical fitness (symptom-limited exercise test, 6MWT, sit-to-stand, half-squat, and arm curl), Quality of life (Laval questionnaire), PA barriers (physical exercise belief questionnaire), anthropometric parameters (BMI, weight), body composition, blood pressure and HR, and satisfaction	12 weeks pre-op
Bond et al. (2015) [59]	Randomized controlled trial (RCT)	N = 75 (analysis); BMI: 45.0 ± 6.5; age: 46.0 (8.9); female: 86.7% (sample N = 36: age 47.0 (8.2), female 86.1%)	Physical activity counseling (PAI) individual face-to-face with behavioral strategies (e.g., self-monitoring, goal setting); 6 weeks; weekly; counseling	Standard pre-surgical care (SC)	Physical activity (objective MVPA, bout-related and total), barriers, self-efficacy, and weight	6 weeks pre-op + post-op follow-up
Creel et al. (2016) [60]	Randomized controlled trial (RCT)	N = 150 randomized, N = 107 (ITT analysis); BMI: NR; age: 43.2 ± 11.2 (randomized); female: 84.1% (ITT)	Counseling (collaborative vs. prescriptive) vs. pedometer use; focus on 6 weeks pre-op; frequency NR; supervision NR	Standard care (SC)	Physical activity (accelerometry, self-report), Physical capacity (submaximal exercise test—METs), sedentary and light PA %, and steps	6 weeks pre-op + 2, 4, and 6 months post-op
Funderburk et al. (2010) [65]	Randomized controlled pilot trial (quasi-experimental pre-post design)	N = 7 (4 intervention, 3 control). Individuals with morbid obesity (BMI > 40). Age: 28–54 years (mean ± SD: intervention: 37.25 ± 5.6; control: 49.3 ± 3.8). % female: 57% (4 females total: 3 control and 1 intervention)	Aquatic therapy (warm-up, strength/endurance exercise). Duration: 12 weeks. Frequency: 2 sessions/week (1 h/session). Supervision: supervised by aquatic leaders.	Continued normal routine (no intervention)	Health-related quality of life (SF-36v2), depression (Beck Depression Inventory, BDI), adjustment to obesity (Obesity Adjustment Scale, OAS), physical status (weight, blood pressure, 6-min walk test [6MWT], and rate of perceived exertion [RPE]).	2 weeks (pre- and post-intervention assessments)
Garcia-Delgado et al. (2021) [61]	RCT protocol and controlled pilot study	N = 15 randomized (pilot: Int = 7, Cont = 8); BMI: ~46.7 (Int 44.7 ± 4.5, Cont 48.5 ± 6.7); age: ~39.5 (Int 38 ± 10, Cont 41 ± 10); female: 14/15 (93.3%)	Physical conditioning + respiratory physiotherapy + standard group intervention; minimum 4 months; 2x/week (inferred from context); supervised (inferred from authors)	Standard treatment (education + cognitive behavioral therapy)	Preoperative weight-loss, functional capacity (6MWT, handgrip strength), quality of Life (EQ–5D–5L, HAD Scale), physical activity (IPAQ), adherence, body composition (weight, BMI, FM, and PA angle), and postoperative complications (Clavien–Dindo)	Minimum 4 months pre-op + 30 days post-op
Li et al. (2013) [62]	Three-arm unblinded pilot randomized controlled trial (RCT)	N: 22 patients (8 home-based, 7 gym-based, and 7 non-intervention). BMI: 47 ± 6 kg/m^2^ (similar across groups). Age: Similar across groups (exact values not reported); % female: not reported.	Home-based group: aerobic + resistance training. Duration: 2 months preoperatively. Frequency: 3 × 30-min aerobic + 2 × resistance sessions/week. Supervision: unsupervised. Gym-based group: Same type, duration, and frequency as home-based. Supervision: supervised by trainer.	Non-intervention group: received standard care without exercise program	Primary: percent excess weight loss (%EWL), maximal aerobic capacity (VO_2_max) pre- and post-surgery. Secondary: Not explicitly detailed beyond %EWL and VO_2_max.	Baseline pre-surgery (2 months post-randomization); 10 weeks post-surgery
Marc-Hernandez et al. (2019) [66]	Controlled pilot study (non-randomized)	N = 23 enrolled (EG = 12, CG = 11); BMI: ~44.5 (EG 47.5 ± 7.1, CG 41.5 ± 2.7); age: ~45.4 (EG 45.4 ± 8.2, CG 45.4 ± 8.2); female: 83% (19/23)	Monitored and supervised exercise program (aerobic + resistance, progressive); 12 weeks; 3x/week; monitored and supervised	Usual pre-surgical care + advice to follow active lifestyle	Body composition (weight, BMI, FM, and FFM), anthropometric measures (waist/hip circ., visceral fat), physical fitness (VO_2_peak, strength), and cardiometabolic risk factors (glucose, TC, and LDL-C)	12 weeks pre-op
Marcon et al. (2016) [63]	Randomized controlled trial (RCT)	N = 57 analyzed; BMI: NR; age: NR; female: ~96.5% (55/57), per group: CONTROL 100%, EXER 95.5%, and EXER + CBT 94.1%	Exercise (EXER) vs. exercise + cognitive behavioral therapy (EXER + CBT); 18–21 weeks; 2–3x/week; supervised	Control (usual care)	Functional capacity (VO_2_max estimated, 6MWT), weight, resting heart rate (HRrest), blood pressure (SBP), HDL, total cholesterol, triglycerides, and glucose	18–21 weeks pre-op
Parikh et al. (2012) [64]	Randomized controlled trial (RCT)	N = 55 enrolled, N = 29/26 ITT, N = 10/13 completers; BMI: ~45.4 (ITT baseline MSWM 45.82, UC 44.99); age: ~45.15 (ITT baseline); female: 100% (55/55)	Medically supervised weight management (MSWM) (combined individual/group monthly visits for analysis); 6 months; monthly; medically supervised	Usual care (UC)	BMI, physical activity, adherence, eating behavior, patient activation, weight loss (implied by BMI changes)	6 months pre-op + 6 months post-op
Pico-Sirvent et al. (2019) [67]	Controlled pilot study (non-randomized)	N = 6 (EG = 3, CG = 3); BMI: 38.78 ± 1.18; age: 38.17 ± 12.06; female: 5/6 (83.3%) (EG = 2/3, CG = 3/3)	Combined exercise training program (ETP) (HIIT + resistance, progressive); 6 months; 3x/week; supervised	Control group (followed usual medical indications)	Body composition (weight, BMI, FM, FFM, and visceral fat), physical fitness (VO_2_peak), cardiometabolic health (SBP, DBP, and HRrest), anthropometric measures (waist/hip circumference	6 months pre-op
Pico-Sirvent et al. (2022) [68]	Controlled study (non-randomized)	N = 20 (EG = 10, CG = 10); BMI: NR (weight given: EG 125.3 ± 13.9, CG 115.8 ± 15.1); age: ~42.5 (EG 43 ± 5, CG 42 ± 9); female: 100% (20/20)	Combined aerobic training (at Fatmax) + resistance (low intensity); 12 weeks; frequency NR; supervised	Conventional care	Body composition (weight, FM, FFM, visceral fat—via bioimpedance), fat oxidation (resting RFO, exercise MFO), resting metabolic rate (RMR), physical fitness (VO_2_peak, strength (POpeak)), RCHO, VT2, and Fatmax intensity/HR/VO_2_	12 weeks pre-op
Still et al. (2007) [69]	Cohort study	N = 884; BMI: 51.3 ± 8.0; age: 45 ± 10; female: 78%	Standard multidisciplinary preoperative program (medical, psychological, nutritional, surgical, education, behavioral modules, support groups, and attempt to lose 10% EBW)	Patients within the program observed (comparison based on preoperative weight loss groups)	Hospital length of stay, postoperative complications, weight/BMI changes, preoperative weight loss, and % EBW loss at 6–12 months post-op based on pre-op weight loss groups	Up to 27 months post-op (data shown for 6 and 12 mos)

Abbreviations: 6MWT: 6-min walk test; BDI: Beck Depression Inventory; BMI: body mass index; CBT: cognitive behavioral therapy; CG: control group; CSEP: core stabilization exercise program; EG: exercise group; ETP: exercise training program; Exp: experimental group; FM: fat mass; FFM: fat-free mass; FSS: fatigue severity scale; Func Cap: functional capacity; GXT: graded exercise test; hR: heart rate; IMT: inspiratory muscle training; IPAQ: International Physical Activity Questionnaire; IQR: interquartile range; METs: metabolic equivalents; mos: months; MVPA: moderate-to-vigorous physical activity; N: number of participants; NR: not reported; NSEG: non-supervised exercise group; OAS: Overall Anxiety Severity and Impairment Scale; OSQOL: obesity-specific quality of life; PA: physical activity; PAI: physical activity intervention; PreSET: pre-surgical exercise training; QoL: quality of life; RCHO: resting carbohydrate oxidation; RCT: randomized controlled trial; RFO: resting fat oxidation; RMR: resting metabolic rate; SC: standard care; SCD: standard care diet/counseling; SD: standard deviation; SEG: supervised exercise group; SF-36 PF: Short-form 36 Physical Functioning subscale; UC: usual care; VO_2_max: maximal oxygen uptake; Wt: weight; wks: weeks; yrs: years.

### 3.3. Risk of Bias Assessment of Included Studies

#### 3.3.1. Risk of Bias in Included Randomized Controlled Trials

The assessment of risk of bias in randomized controlled trials showed variation in methodological quality across different areas. Most studies demonstrated a low risk of bias in random sequence generation, with seven studies explicitly reporting adequate randomization procedures [29,57,58,59,60,61,63]. Allocation concealment was adequately described in five studies using appropriate concealment methods [29,58,60,61,63], while four studies provided insufficient details [57,59,62,64]. Blinding of participants and personnel presented the greatest challenge, with all nine studies showing high risk of performance bias [29,57,58,59,60,61,62,63,64] due to participants’ awareness of exercise intervention assignments, which is inherent to the nature of physical activity interventions. For outcome assessment blinding, three studies demonstrated low risk by explicitly blinding outcome assessors [57,59,60], two received high-risk ratings for lack of blinding [58,60], and five studies had unclear risk due to insufficient information [29,61,62,63,64]. Most studies showed low risk of attrition bias, with six studies adequately addressing incomplete outcome data [57,58,60,61,63,70], while two studies were rated high-risk due to significant dropout rates or inadequate handling of missing data [61,64]. Regarding other biases, four studies showed low risk [29,57,61,70], while three had unclear risk due to insufficient reporting [58,60,63], and two received high-risk ratings for additional methodological concerns [62,64] (Figures S1 and S2).

#### 3.3.2. Risk of Bias in Included Non-Randomized Studies

The risk of bias in the six non-randomized studies was assessed using the ROBINS-I tool [37]. Three studies were identified as having a critical risk of bias [65,67,69], while three were identified as having a serious risk [28,66,68]. Confounding was identified as the most problematic area, with all studies demonstrating serious to critical risk due to non-randomized group allocation and inadequate control for baseline differences between groups. Selection bias varied across studies, with four studies demonstrating serious risk [28,65,67,69], one showing critical risk [68], and one with moderate risk [66]. Classification of interventions was consistently rated as low risk across all studies. Deviations from intended interventions, missing data, and outcome measures were predominantly low to moderate risk, with most studies showing adequate adherence to intervention protocols and appropriate outcome measurement strategies (Figures S3 and S4).

### 3.4. Quantitative Meta-Analysis

A total of 9 studies with 183 participants were included for the analysis of weight change, 7 studies with 165 participants were included for the analysis of BMI change, 4 studies with 129 participants were included for the analysis of six-minute walk test change, 3 studies with 72 participants were included for the analysis of VO_2_ peak change, and 1 study with 14 participants was included for the analysis of the length of hospital stay (Table 2). Additional anthropometric measures, including waist circumference, hip circumference, visceral fat, and waist-to-hip ratio, were reported in a subset of studies. However, these outcomes were reported with insufficient frequency and statistical detail to permit meta-analysis.

The interventions were categorized into three main types, namely supervised combined exercise programs, such as incorporating aerobic and resistance training; specific supervised exercise programs, such as those focused on particular exercise modalities; and multimodal interventions that combined exercise with other preoperative strategies, such as nutritional support or inspiratory muscle training [28,29,57,58,61,62,63,64,65,66,67,68].

#### 3.4.1. Weight Change

**Figure 2:**
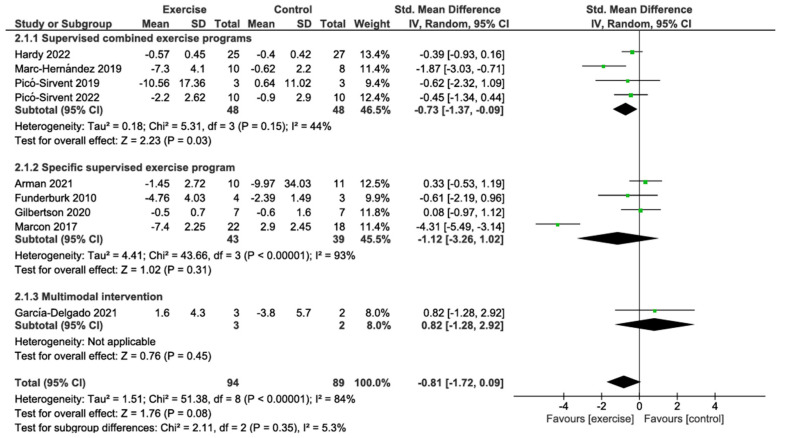
Meta-analysis of weight change [28,29,57,61,63,65,66,67,68].

The primary analysis using a random effects model showed that preoperative exercise interventions were not significantly associated with weight reduction compared with the control group (SMD −0.81; 95% CI: −1.72 to 0.09; *p* = 0.08), with substantial heterogeneity between studies (I^2^ = 84%). Subgroup analysis revealed no significant differences between intervention types (*p* = 0.35; I^2^ = 5.3%). Supervised combined exercise programs showed a significant reduction in weight (SMD −0.73; 95% CI: −1.37 to −0.09; *p* = 0.03; I^2^ = 44%), based on 4 studies with 48 patients. This represents a medium effect size according to Cohen’s conventions. Conversely, specific supervised exercise programs showed a non-significant trend towards weight reduction (SMD −1.12; 95% CI: −3.26 to 1.02; *p* = 0.31; I^2^ = 93%), based on 4 studies with 43 patients. The multimodal intervention subgroup, comprising a single study with three patients, showed no significant effect (SMD 0.82; 95% CI: −1.28 to 2.92; *p* = 0.45). According to the GRADE approach, the certainty of the evidence for this outcome was rated as very low, primarily due to risk of bias from lack of blinding, substantial heterogeneity, indirectness from indirect estimation of standard deviations, and imprecision from small sample sizes (Figure 2; Table 2).

#### 3.4.2. BMI Change

**Figure 3:**
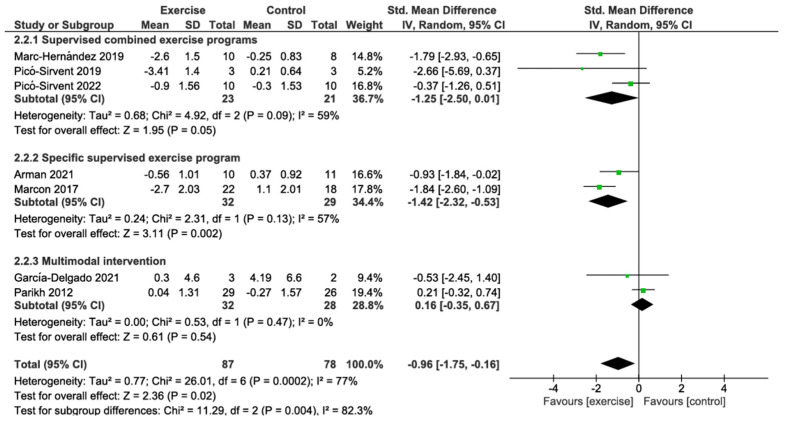
Meta-analysis of BMI change [57,61,63,64,66,67,68].

The primary analysis using a random effects model showed that preoperative exercise interventions were significantly associated with BMI reduction compared with the control group (SMD −0.96; 95% CI: −1.75 to −0.16; *p* = 0.02), with substantial heterogeneity between studies (I^2^ = 77%). Subgroup analysis revealed significant differences between intervention types (*p* = 0.004; I^2^ = 82.3%). Supervised combined exercise programs showed a borderline significant reduction in BMI (SMD −1.25; 95% CI: −2.50 to 0.01; *p* = 0.05; I^2^ = 59%), based on 3 studies with 23 patients. This represents a large effect size according to Cohen’s conventions. Specific supervised exercise programs showed a significant reduction in BMI (SMD −1.42; 95% CI: −2.32 to −0.53; *p* = 0.002; I^2^ = 57%), based on 2 studies with 32 patients, also representing a large effect size. Conversely, multimodal interventions showed no significant effect (SMD 0.16; 95% CI: −0.35 to 0.67; *p* = 0.54; I^2^ = 0%), based on 2 studies with 32 patients. According to the GRADE approach, the certainty of the evidence for this outcome was rated as very low, primarily due to risk of bias from lack of blinding, substantial heterogeneity, indirectness from indirect estimation of standard deviations, and imprecision from small sample sizes (Figure 3; Table 2).

#### 3.4.3. Six-Minute Walk Test Change

**Figure 4:**
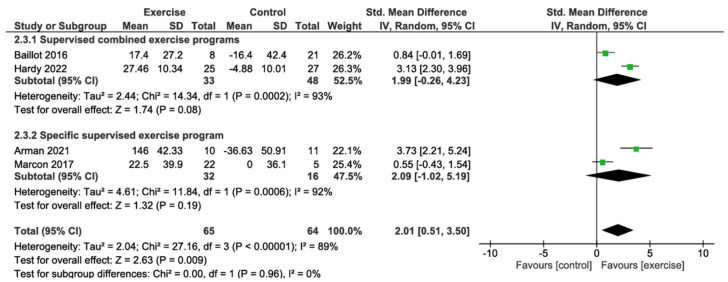
Meta-analysis of six-minute walk test change [29,57,58,63].

The primary analysis using a random effects model showed that preoperative exercise interventions were significantly associated with improvements in 6-min walk test distance compared with the control group (SMD 2.01; 95% CI: 0.51 to 3.50; *p* = 0.009), with substantial heterogeneity between studies (I^2^ = 89%). Subgroup analysis revealed no significant differences between intervention types (*p* = 0.96; I^2^ = 0%). Supervised combined exercise programs showed a non-significant trend towards improvement in walking distance (SMD 1.99; 95% CI: −0.26 to 4.23; *p* = 0.08; I^2^ = 93%), based on 2 studies with 33 patients. This represents a large effect size according to Cohen’s conventions. Specific supervised exercise programs showed a non-significant improvement (SMD 2.09; 95% CI: −1.02 to 5.19; *p* = 0.19; I^2^ = 92%), based on 2 studies with 32 patients, also representing a large effect size. According to the GRADE approach, the certainty of the evidence for this outcome was rated as very low, primarily due to risk of bias from lack of blinding, substantial heterogeneity, indirectness from indirect estimation of standard deviations, and imprecision from small sample sizes (Figure 4; Table 2).

#### 3.4.4. VO_2_ Peak Change

**Figure 5:**
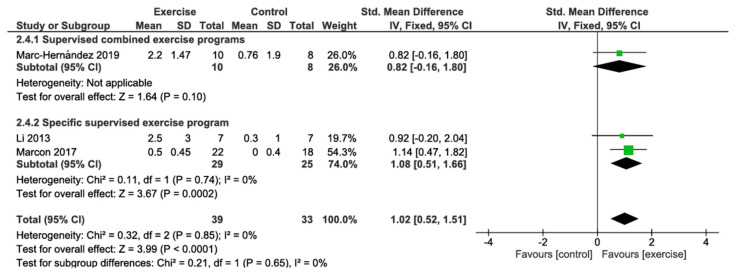
Meta-analysis of six-minute VO_2_ peak change [62,63,66].

The primary analysis using a fixed effects model showed that preoperative exercise interventions were significantly associated with improvements in VO_2_ peak compared with the control group (SMD 1.02; 95% CI: 0.52 to 1.51; *p* < 0.0001), with minimal heterogeneity between studies (I^2^ = 0%). Subgroup analysis revealed no significant differences between intervention types (*p* = 0.65; I^2^ = 0%). Supervised combined exercise programs showed a non-significant trend towards improvement in VO_2_ peak (SMD 0.82; 95% CI: −0.16 to 1.80; *p* = 0.10), based on 1 study with 10 patients. This represents a large effect size according to Cohen’s conventions. Specific supervised exercise programs showed a significant improvement (SMD 1.08; 95% CI: 0.51 to 1.66; *p* = 0.0002; I^2^ = 0%), based on 2 studies with 29 patients, also representing a large effect size. According to the GRADE approach, the certainty of the evidence for this outcome was rated as very low, primarily due to risk of bias from lack of blinding, indirectness from indirect estimation of standard deviations, and imprecision from small sample sizes (Figure 5; Table 2).

#### 3.4.5. Length Hospital Stay (Days)

The primary analysis using a fixed effects model showed that preoperative exercise interventions were significantly associated with reduced length of hospital stay compared with the control group (MD −0.64 days; 95% CI: −0.86 to −0.42; *p* < 0.00001), based on a single study with 14 patients [28]. No heterogeneity assessment was applicable given the single study design. According to the GRADE approach, the certainty of the evidence for this outcome was rated as low, primarily due to risk of bias from lack of blinding and imprecision from the small sample size (Table 2).

## 4. Discussion

This systematic review examined the effectiveness of preoperative exercise interventions on outcomes in patients undergoing metabolic and bariatric surgery. Our focus on surgical candidates represents a distinct population from general obesity exercise intervention studies, as these individuals face imminent surgical stress and have specific optimization needs. Unlike exercise interventions in the general population with obesity, which primarily target weight loss and metabolic improvement, preoperative exercise in bariatric surgery candidates aims to enhance physiological reserve, improve surgical safety, and potentially optimize postoperative recovery. This distinction is important because the limited time available in the preoperative period and the specific goal of surgical preparation mean that exercise prescription and outcome assessment require a unique approach.

The findings evindenced mixed but largely beneficial effects of structured exercise programs on key preoperative outcomes, with notable improvements in functional capacity measures and modest effects on body composition parameters. The meta-analysis revealed significant improvements in cardiorespiratory fitness, as evidenced by large effect sizes for both six-minute walk test distance and VO_2_ peak. These findings align with previous research demonstrating the physiological benefits of preoperative conditioning programs in surgical populations. The improvement in functional capacity is particularly relevant given that poor cardiorespiratory fitness has been identified as a risk factor for postoperative complications in bariatric surgery patients. The consistency of these findings across different exercise modalities suggests that structured preoperative exercise, regardless of the specific protocol, can effectively enhance aerobic capacity in this population.

The results for body composition changes present a more nuanced picture. While the overall effect on weight change was not statistically significant, the meta-analysis revealed a significant reduction in BMI, with both supervised exercise modalities demonstrating large effect sizes. This apparent discrepancy may reflect differences in body composition changes, where exercise interventions may promote fat loss while preserving or increasing lean muscle mass, resulting in BMI reduction without proportional weight loss. The finding that supervised combined exercise programs showed the most consistent effects on both weight and BMI suggests that multimodal exercise approaches may be superior to single-modality interventions.

Subgroup analyses revealed important differences between intervention types. Supervised combined exercise programs, which incorporated both aerobic and resistance training components, demonstrated more consistent beneficial effects across outcomes compared to specific supervised exercise programs or multimodal interventions. This finding supports the theoretical rationale for comprehensive exercise programming that addresses multiple aspects of physical fitness simultaneously. The superior performance of supervised versus unsupervised interventions underscores the importance of professional guidance and monitoring in achieving optimal preoperative conditioning. The absence of funding from exercise equipment manufacturers or bariatric surgery device companies across all studies reduces concerns about commercial bias, though most studies relied on public or institutional funding, which may influence the types of interventions studied.

The significant reduction in length of hospital stay observed in the single study reporting this outcome is clinically meaningful and economically relevant. However, this finding must be interpreted cautiously given the limited evidence base. The potential for exercise interventions to reduce healthcare utilization warrants further investigation, particularly given the growing emphasis on value-based care in bariatric surgery programs.

To contrast our findings with existing evidence, we examined recent systematic reviews and meta-analyses that evaluated the effects of preoperative exercise in candidates for bariatric surgery. In the domain of functional capacity, our review identified consistent improvements associated with exercise-based interventions. Regarding functional capacity, our review identified consistent improvements following preoperative exercise interventions. A recent meta-analysis by Ghannadi et al. [33] evaluated the effect of pre-surgical exercise training on cardiorespiratory fitness. This study, which synthesized data from ten trials, reported statistically significant standardized mean differences for peak VO_2_, as well as weighted mean differences for the six-minute walk test (6MWT) distance—both indicating enhancements in cardiorespiratory fitness. These findings are strongly aligned with our own results, reinforcing the evidence that preoperative exercise contributes meaningfully to improved functional capacity in bariatric surgery candidates. Similarly, a systematic review and meta-analysis by Durey et al. [32] also found modest gains in VO_2_max following preoperative exercise interventions, albeit based on a limited number of randomized controlled trials. The consistency in the direction of effect across these reviews supports the existence of a true benefit of preoperative exercise on physical fitness.

Our review also demonstrated a significant increase in physical activity levels and a reduction in sedentary behavior. A systematic review by Jabbour et al. [19] synthesized evidence on preoperative physical activity levels and bariatric surgery outcomes, highlighting the beneficial role of structured exercise programs prior to surgery. Their findings suggested that patients with higher levels of preoperative physical activity experienced better outcomes across anthropometric, cardiometabolic, and functional parameters. Our observation that preoperative exercise interventions increase physical activity supports a plausible mechanism for the improvements reported by Jabbour et al. [19].

In terms of postoperative complications, our review identified a reduction in one multimodal intervention study. However, a systematic review and meta-analysis by Herrera-Santelices et al. [71], which specifically evaluated supervised physical exercise as prehabilitation in bariatric surgery candidates, found no evidence of surgical outcome improvements in the included studies. This discrepancy may indicate that multimodal prehabilitation programs—combining exercise with nutritional support or inspiratory muscle training—could be more effective in reducing postoperative complications compared to isolated exercise interventions. The lack of data on surgical outcomes in Herrera-Santelices et al. highlights a critical gap and underscores the need for further targeted investigation [71].

With respect to body weight and BMI, our findings suggest minimal preoperative changes associated with exercise alone. Lodewijks et al. [72] conducted a systematic review on the impact of preoperative programs on weight loss, reporting that while interventions including diet, behavioral modification, and occasionally exercise generally facilitated pre-surgical weight reduction, only one study focusing solely on exercise showed a significant effect on postoperative weight loss. This supports our conclusion that exercise as a standalone intervention may not suffice to induce meaningful preoperative weight loss in this population.

Concerning patient-reported outcomes, such as quality of life (QoL), our review reported significant improvements. Herrera-Santelices et al. [71] similarly identified a positive effect of exercise on overall QoL scores among bariatric surgery candidates, strengthening the evidence for the beneficial impact of preoperative exercise on this important outcome. In contrast, Ghannadi et al. [33] did not observe a statistically significant effect of preoperative exercise on QoL. This divergence could be attributed to differences in the QoL measurement instruments used, the characteristics of the exercise interventions, or the timing of post-intervention assessments.

Lastly, our review found limited evidence regarding metabolic parameters. Nevertheless, Jabbour et al. [19] reported an association between higher preoperative physical activity and improvements in cardiometabolic markers. Although direct evidence from our included studies was scarce, the link proposed by Jabbour et al. [19] suggests that increased physical activity induced by preoperative exercise may indirectly contribute to metabolic benefits, warranting further investigation specifically focused on this pathway.

Several methodological considerations limit the interpretation of these findings. The substantial heterogeneity observed across most outcomes reflects the diversity in exercise protocols, patient populations, and outcome measurement approaches among included studies. While this heterogeneity was addressed through subgroup analyses and the use of random effects models, it indicates a need for greater standardization in preoperative exercise research.

An important limitation is the way in which pre-bariatric exercise preconditioning was evaluated. Although VO_2_ peak and the six-minute walk test were used as the main measures of cardiorespiratory fitness, these assessments may not accurately reflect the multifaceted nature of physical conditioning relevant to surgical outcomes. The considerable variation in exercise intervention characteristics also makes interpretation difficult. Exercise duration ranged from 30 days to 6 months, with the frequency of sessions per week varying from two to four. Session duration ranged from 30 to 80 min, with many studies employing progressive increases throughout the intervention period. This heterogeneity in exercise prescription parameters, including duration, frequency, and intensity, makes it difficult to determine the most effective preconditioning protocols.

Furthermore, the relative importance of different types of exercise remains unclear. Although our subgroup analyses showed that supervised programmes combining aerobic and resistance training had more consistent beneficial effects across outcomes than single-modality interventions, the optimal balance between these components has not been determined. It is notable that only eight studies implemented combined aerobic and resistance training, while four focused exclusively on aerobic exercise. Given the limited number of studies focusing primarily on resistance training, the potential superiority of strength training over aerobic exercise for improving surgical outcomes could not be adequately assessed. This represents a critical knowledge gap, given that muscle strength and lean mass preservation are particularly important for postoperative recovery and long-term metabolic outcomes in bariatric surgery patients.

The predominance of small studies with methodological limitations, as reflected in the uniformly low to very low certainty of evidence ratings, emphasizes the need for larger, well-designed randomized controlled trials. The indirect estimation of standard deviations in many studies introduced additional uncertainty into the meta-analyses, necessitating the use of standardized mean differences for most continuous outcomes. While this approach enabled the pooling of studies with incomplete statistical reporting, it may have obscured clinically meaningful differences in absolute terms. Future research should prioritize complete and standardized statistical reporting to facilitate more precise meta-analytic synthesis. An additional limitation relates to the mixing of study designs in our meta-analyses. Although we planned to conduct separate analyses for RCTs and non-randomized studies, the small number of studies per outcome made this approach unfeasible.

However, the evidence supporting improvements in functional capacity provides a strong rationale for implementing structured preoperative exercise programs in bariatric surgery candidates. Despite generally minimal changes in preoperative weight with exercise alone, the consistent benefits in functional capacity, increased habitual physical activity, and potential reduction in complications suggest that exercise can play a crucial role in optimizing patient preparation for surgery. These benefits are particularly important given that patients with obesity often present with higher surgical risk and poorer postoperative recovery.

Healthcare professionals attending to patients with obesity undergoing bariatric surgery, including physicians, bariatric surgeons, and physiotherapists, should consider preoperative exercise as a valuable intervention. The implementation of such programs should prioritize supervised, multimodal approaches while acknowledging the need for further high-quality research to establish optimal protocols and expand the evidence base for surgical and long-term outcomes.

It is important to distinguish our findings from those of the broader literature on exercise interventions for people with obesity. While obesity exercise studies generally focus on long-term weight loss, metabolic improvements, and cardiovascular risk reduction over extended periods, our review specifically addresses the unique context of surgical preparation. The preoperative period presents both opportunities and constraints: the defined timeframe before surgery (ranging from 30 days to 6 months in the studies included in our review) creates an urgent need for optimization, but limits the potential for substantial weight loss through exercise alone. Furthermore, the primary goal shifts from weight reduction to enhancing functional capacity and physiological resilience in order to withstand surgical stress. This explains why our meta-analysis found significant improvements in functional measures (6MWT and VO_2_ peak) with large effect sizes, while changes in weight and BMI were more modest. These results imply that preoperative exercise serves a different purpose to that of traditional weight management programmes, with a focus on surgical readiness rather than primary weight loss.

Future research priorities should include large-scale randomized controlled trials with standardized exercise protocols and comprehensive outcome assessment, including surgical complications, long-term functional outcomes, and economic evaluations. Investigation of optimal exercise duration, intensity, and modality combinations is needed to refine clinical recommendations. Additionally, research examining the interaction between preoperative exercise and other prehabilitation interventions could provide valuable insights for comprehensive preoperative care protocols. Future studies should strive to standardize measured outcomes, including detailed surgical parameters and comprehensive metabolic evaluations, to facilitate comparison between studies and enable more robust meta-analyses. Greater research is also required to elucidate the underlying metabolic and molecular mechanisms of preoperative exercise benefits in this population, as well as to investigate the long-term effects of preoperative exercise on sustained postoperative weight loss and long-term physical activity maintenance.

## 5. Conclusions

This systematic review provides evidence supporting the beneficial effects of preoperative exercise interventions on functional capacity measures in bariatric surgery candidates. The wide confidence intervals and small sample sizes further limit the clinical interpretability of these findings. While large statistical effect sizes were observed for functional outcomes, the confidence intervals often span from potentially clinically meaningful to minimal effects, reflecting considerable uncertainty about the magnitude of benefit individual patients might expect. While the evidence for body composition changes is mixed and certainty of evidence is low for most outcomes, the consistent improvements in cardiorespiratory fitness support the integration of structured exercise programs into preoperative care pathways. The implementation of such programs should prioritize supervised, multimodal approaches while acknowledging the need for further high-quality research to establish optimal protocols and expand the evidence base for surgical and long-term outcomes.

## Figures and Tables

**Table 2:** Summary of findings table using the GRADE approach.

**Exercise compared to control for patients undergoing metabolic and bariatric surgery**					
**Patient or population:** Patients undergoing metabolic and bariatric surgery. **Intervention:** Exercise. **Comparison:** Control.					
**Outcomes**	**№ of participants** (**studies)** **Follow-up**	**Certainty of the evidence** **(GRADE)**	**Relative effect** **(95% CI)**	**Anticipated absolute effects**	
				**Risk with control**	**Risk difference with exercise**
Weight change (Kg)	183 (9 studies)	⨁◯◯◯ Very low ^a,b,c,d^	-	-	SMD **0.81 lower** (1.72 lower to 0.09 higher)
BMI change (Kg/m^2^)	165 (7 studies)	⨁◯◯◯ Very low ^a,b,c,d^	-	-	SMD **0.96 lower** (1.75 lower to 0.16 lower)
6-min walk test change (m)	129 (4 studies)	⨁◯◯◯ Very low ^a,b,c,d^	-	-	SMD **2.01 higher** (0.51 higher to 3.5 higher)
VO_2_ peak change	72 (3 studies)	⨁◯◯◯ Very low ^a,c,d^	-	-	SMD **1.02 higher** (0.52 higher to 1.51 higher)
Length of hospital stay (days)	14 (1 study)	⨁⨁◯◯ Low ^a,d^	-	The mean length of hospital stay (days) was **2.36** days	MD **0.64 days lower** (0.86 lower to 0.42 lower)

**The risk in the intervention group** (and its 95% confidence interval) is based on the assumed risk in the comparison group and the **relative effect** of the intervention (and its 95% CI). **CI:** confidence interval; **MD:** mean difference; **SMD:** standardized mean difference. **GRADE working group grades of evidence. High certainty** (⨁⨁⨁⨁): We are very confident that the true effect lies close to that of the estimate of the effect. **Moderate certainty** (⨁⨁⨁◯): We are moderately confident in the effect estimate: the true effect is likely to be close to the estimate of the effect, but there is a possibility that it is substantially different. **Low certainty** (⨁⨁◯◯): Our confidence in the effect estimate is limited: the true effect may be substantially different from the estimate of the effect. **Very low certainty** (⨁◯◯◯): We have very little confidence in the effect estimate: the true effect is likely to be substantially different from the estimate of effect. ^a^. The certainty of the evidence was downgraded by one level for risk of bias; the blinding of participants or personnel were high in all included trials. ^b^. The certainty of evidence was downgraded by one level due to inconsistency; the heterogeneity is substantial (I^2^ > 50%). ^c^. The certainty of evidence was downgraded by one level due to indirectness, as in some studies the standard deviation of group means was estimated indirectly. ^d^. The certainty of evidence was downgraded for imprecision, as the studies together had a small sample size.

## Data Availability

The original contributions presented in this study are included in the article/Supplementary Material. Further inquiries can be directed to the corresponding author.

## Supplementary Materials

The following supporting information can be downloaded at: https://www.mdpi.com/article/10.3390/jcm14176170/s1.
