# Supplementary material for: Effects of Preoperative Exercise Interventions in Patients Undergoing Metabolic and Bariatric Surgery: A Systematic Review and Meta-Analysis
Source: J Clin Med. 2025 Sep 1;14(17):6170. doi: 10.3390/jcm14176170 (PMC12429658; doi:10.3390/jcm14176170)
Supplement: Supplementary file 1 [file jcm-14-06170-s001.zip › jcm-3756509-supplementary.pdf]

Table S1. Search strategy performed in the different databases.

Database	Search strategy
Medline/PubMed **(n= 1191)**	(((((((("bariatric surger*"[Title/Abstract]) OR ("metabolic surger*"[Title/Abstract])) OR ("stomach stapling"[Title/Abstract])) OR ("gastroenterostomy"[Title/Abstract])) OR ("gastroplasty"[Title/Abstract])) OR ("gastric bypass"[Title/Abstract])) OR (("bariatric surgery"[MeSH Terms]))) AND ((((("preoperative"[Title/Abstract]) OR ("pre-operative"[Title/Abstract])) OR ("presurg*"[Title/Abstract])) OR ("pre-surg*"[Title/Abstract])) OR ("Preoperative Period"[MeSH Terms] OR "Preoperative Care"[MeSH Terms] OR "Preoperative Exercise"[MeSH Terms]))) AND ((((((((((("pharmacological intervention*"[Title/Abstract]) OR ("pharmacologic action*"[Title/Abstract])) OR ("drug therapy"[Title/Abstract])) OR ("pharmacotherapy"[Title/Abstract])) OR ("glucagon-like peptide-1 receptor agonists"[Title/Abstract])) OR ("GLP-1 receptor agonists"[Title/Abstract])) OR ("orlistat"[Title/Abstract])) OR ("anti-obesity agent*"[Title/Abstract])) OR ("antiobesity agent*"[Title/Abstract])) OR ("weight loss drugs"[Title/Abstract])) OR ((((("pharmacologic actions"[MeSH Terms]) OR ("drug therapy"[MeSH Terms])) OR ("orlistat"[MeSH Terms])) OR ("glucagon-like peptide-1 receptor agonists"[MeSH Terms])) OR ("anti-obesity agents"[MeSH Terms]))) OR ((((((((((("non-pharmacological intervention*"[Title/Abstract]) OR ("non-pharmacological action*"[Title/Abstract])) OR ("diet"[Title/Abstract])) OR ("exercise"[Title/Abstract])) OR ("behavioral therap*"[Title/Abstract])) OR ("behavioral intervention*"[Title/Abstract])) OR ("lifestyle modification*"[Title/Abstract])) OR ("combined approach*"[Title/Abstract])) OR ("gastric balloon*"[Title/Abstract])) OR ("intragastric balloon*"[Title/Abstract])) OR (((("diet, reducing"[MeSH Terms]) OR ("preoperative exercise"[MeSH Terms])) OR ("behavior therapy"[MeSH Terms])) OR ("gastric balloon"[MeSH Terms]))))
Epistemonikos **(n= 487)**	(title:((title:(bariatric surger*) OR abstract:(bariatric surger*)) OR (title:(metabolic surger*) OR abstract:(metabolic surger*)) OR (title:(stomach stapling) OR abstract:(stomach stapling)) OR (title:(gastroenterostomy) OR abstract:(gastroenterostomy)) OR (title:(gastroplasty) OR abstract:(gastroplasty)) OR (title:(gastric bypass) OR abstract:(gastric bypass))) OR abstract:((title:(bariatric surger*) OR abstract:(bariatric surger*)) OR (title:(metabolic surger*) OR abstract:(metabolic surger*)) OR (title:(stomach stapling) OR abstract:(stomach stapling)) OR (title:(gastroenterostomy) OR abstract:(gastroenterostomy)) OR (title:(gastroplasty) OR abstract:(gastroplasty)) OR (title:(gastric bypass) OR abstract:(gastric bypass)))) AND (title:((title:(preoperative) OR abstract:(preoperative)) OR (title:(pre-operative) OR abstract:(pre-operative)) OR (title:(presurg*) OR abstract:(presurg*)) OR (title:(pre-surg*) OR abstract:(pre-surg*))) OR abstract:((title:(preoperative) OR abstract:(preoperative)) OR (title:(pre-operative) OR abstract:(pre-operative)) OR (title:(presurg*) OR abstract:(presurg*)) OR (title:(pre-surg*) OR abstract:(pre-surg*)))) AND (title:((title:((title:(pharmacological intervention*) OR abstract:(pharmacological intervention*)) OR (title:(pharmacologic action*) OR abstract:(pharmacologic action*)) OR (title:(drug therapy) OR abstract:(drug therapy)) OR (title:(pharmacotherapy) OR abstract:(pharmacotherapy)) OR (title:(glucagon-like peptide-1 receptor agonists) OR abstract:(glucagon-like peptide-1 receptor agonists)) OR (title:(GLP-1 receptor agonists) OR abstract:(GLP-1 receptor agonists)) OR (title:(orlistat) OR abstract:(orlistat)) OR (title:(anti-obesity agent*) OR abstract:(anti-obesity agent*)) OR (title:(antiobesity agent*) OR abstract:(antiobesity agent*)) OR (title:(weight loss drugs) OR

	abstract:(weight loss drugs))) OR abstract:((title:(pharmacological intervention*) OR abstract:(pharmacological intervention*)) OR (title:(pharmacologic action*) OR abstract:(pharmacologic action*)) OR (title:(drug therapy) OR abstract:(drug therapy)) OR (title:(pharmacotherapy) OR abstract:(pharmacotherapy)) OR (title:(glucagon-like peptide-1 receptor agonists) OR abstract:(glucagon-like peptide-1 receptor agonists)) OR (title:(GLP-1 receptor agonists) OR abstract:(GLP-1 receptor agonists)) OR (title:(orlistat) OR abstract:(orlistat)) OR (title:(anti-obesity agent*) OR abstract:(anti-obesity agent*)) OR (title:(antiobesity agent*) OR abstract:(antiobesity agent*)) OR (title:(weight loss drugs) OR abstract:(weight loss drugs)))) OR (title:((title:(non-pharmacological intervention*) OR abstract:(non-pharmacological intervention*)) OR (title:(non-pharmacological action*) OR abstract:(non-pharmacological action*)) OR (title:(diet) OR abstract:(diet)) OR (title:(exercise) OR abstract:(exercise)) OR (title:(behavioral therap*) OR abstract:(behavioral therap*)) OR (title:(behavioral intervention*) OR abstract:(behavioral intervention*)) OR (title:(lifestyle modification*) OR abstract:(lifestyle modification*)) OR (title:(combined approach*) OR abstract:(combined approach*)) OR (title:(gastric balloon*) OR abstract:(gastric balloon*)) OR (title:(intragastric balloon*) OR abstract:(intragastric balloon*))) OR abstract:((title:(non-pharmacological intervention*) OR abstract:(non-pharmacological intervention*)) OR (title:(non-pharmacological action*) OR abstract:(non-pharmacological action*)) OR (title:(diet) OR abstract:(diet)) OR (title:(exercise) OR abstract:(exercise)) OR (title:(behavioral therap*) OR abstract:(behavioral therap*)) OR (title:(behavioral intervention*) OR abstract:(behavioral intervention*)) OR (title:(lifestyle modification*) OR abstract:(lifestyle modification*)) OR (title:(combined approach*) OR abstract:(combined approach*)) OR (title:(gastric balloon*) OR abstract:(gastric balloon*)) OR (title:(intragastric balloon*) OR abstract:(intragastric balloon*))))) OR abstract:((title:((title:(pharmacological intervention*) OR abstract:(pharmacological intervention*)) OR (title:(pharmacologic action*) OR abstract:(pharmacologic action*)) OR (title:(drug therapy) OR abstract:(drug therapy)) OR (title:(pharmacotherapy) OR abstract:(pharmacotherapy)) OR (title:(glucagon-like peptide-1 receptor agonists) OR abstract:(glucagon-like peptide-1 receptor agonists)) OR (title:(GLP-1 receptor agonists) OR abstract:(GLP-1 receptor agonists)) OR (title:(orlistat) OR abstract:(orlistat)) OR (title:(anti-obesity agent*) OR abstract:(anti-obesity agent*)) OR (title:(antiobesity agent*) OR abstract:(antiobesity agent*)) OR (title:(weight loss drugs) OR abstract:(weight loss drugs))) OR abstract:((title:(pharmacological intervention*) OR abstract:(pharmacological intervention*)) OR (title:(pharmacologic action*) OR abstract:(pharmacologic action*)) OR (title:(drug therapy) OR abstract:(drug therapy)) OR (title:(pharmacotherapy) OR abstract:(pharmacotherapy)) OR (title:(glucagon-like peptide-1 receptor agonists) OR abstract:(glucagon-like peptide-1 receptor agonists)) OR (title:(GLP-1 receptor agonists) OR abstract:(GLP-1 receptor agonists)) OR (title:(orlistat) OR abstract:(orlistat)) OR (title:(anti-obesity agent*) OR abstract:(anti-obesity agent*)) OR (title:(antiobesity agent*) OR abstract:(antiobesity agent*)) OR (title:(weight loss drugs) OR abstract:(weight loss drugs)))) OR (title:((title:(non-pharmacological intervention*) OR abstract:(non-pharmacological intervention*)) OR (title:(non-pharmacological action*) OR abstract:(non-pharmacological action*)) OR (title:(diet) OR abstract:(diet)) OR (title:(exercise) OR abstract:(exercise)) OR (title:(behavioral therap*) OR abstract:(behavioral therap*)) OR (title:(behavioral intervention*) OR abstract:(behavioral intervention*)) OR (title:(lifestyle modification*) OR abstract:(lifestyle modification*)) OR (title:(combined approach*) OR abstract:(combined

	approach*)) OR (title:(gastric balloon*) OR abstract:(gastric balloon*)) OR (title:(intragastric balloon*) OR abstract:(intragastric balloon*))) OR abstract:((title:(non-pharmacological intervention*) OR abstract:(non-pharmacological intervention*)) OR (title:(non-pharmacological action*) OR abstract:(non-pharmacological action*)) OR (title:(diet) OR abstract:(diet)) OR (title:(exercise) OR abstract:(exercise)) OR (title:(behavioral therap*) OR abstract:(behavioral therap*)) OR (title:(behavioral intervention*) OR abstract:(behavioral intervention*)) OR (title:(lifestyle modification*) OR abstract:(lifestyle modification*)) OR (title:(combined approach*) OR abstract:(combined approach*)) OR (title:(gastric balloon*) OR abstract:(gastric balloon*)) OR (title:(intragastric balloon*) OR abstract:(intragastric balloon*))))))
Cochrane CENTRAL<br>**(n = 775)**	ID Search Hits<br>#1 (bariatric surger*):ti,ab,kw OR (metabolic surger*):ti,ab,kw OR (gastric bypass):ti,ab,kw OR (gastroenterostomy):ti,ab,kw OR (gastroplasty):ti,ab,kw 8775<br>#2 MeSH descriptor: [Bariatric Surgery] explode all trees1874<br>#3 #1 OR #2 8927<br>#4 (pre-operative):ti,ab,kw OR (presurg*):ti,ab,kw OR (pre-surg*):ti,ab,kw OR (preoperative):ti,ab,kw 52388<br>#5 MeSH descriptor: [Preoperative Period] explode all trees 521<br>#6 MeSH descriptor: [Preoperative Care] explode all trees 7304<br>#7 MeSH descriptor: [Preoperative Exercise] explode all trees 140<br>#8 #4 OR #5 OR #6 OR #7 53926<br>#9 #3 AND #8 1456<br>#10 (pharmacological intervention*):ti,ab,kw OR (pharmacologic action*):ti,ab,kw OR (drug therapy):ti,ab,kw OR (pharmacotherapy):ti,ab,kw OR (weight loss drugs):ti,ab,kw 579760<br>#11 (glucagon-like peptide-1 receptor agonists):ti,ab,kw OR (GLP-1 receptor agonists):ti,ab,kw OR (orlistat):ti,ab,kw OR (anti-obesity agent*):ti,ab,kw OR (antiobesity agent*):ti,ab,kw 2234<br>#12 MeSH descriptor: [Pharmacologic Actions] explode all trees 320599<br>#13 MeSH descriptor: [Drug Therapy] explode all trees 187621<br>#14 MeSH descriptor: [Orlistat] explode all trees 350<br>#15 MeSH descriptor: [Glucagon-Like Peptide-1 Receptor Agonists] explode all trees 36<br>#16 MeSH descriptor: [Anti-Obesity Agents] explode all trees 1059<br>#17 #10 OR #11 OR #12 OR #13 OR #14 OR #15 OR #16 708162<br>#18 (non-pharmacological intervention*):ti,ab,kw OR (non-pharmacological action*):ti,ab,kw OR (diet):ti,ab,kw OR (exercise):ti,ab,kw OR (behavioral therap*):ti,ab,kw 250183<br>#19 (behavioral intervention*):ti,ab,kw OR (lifestyle modification*):ti,ab,kw OR (combined approach*):ti,ab,kw OR (gastric balloon*):ti,ab,kw OR (intragastric balloon*):ti,ab,kw 66082<br>#20 MeSH descriptor: [Diet, Reducing] explode all trees 2503<br>#21 MeSH descriptor: [Preoperative Exercise] explode all trees 140<br>#22 MeSH descriptor: [Behavior Therapy] explode all trees 25737<br>#23 MeSH descriptor: [Gastric Balloon] explode all trees 75<br>#24 #18 OR 19 OR #20 OR #21 OR #22 OR #23 467991<br>#25 #17 OR #24 1022506<br>#26 #9 AND #25 775
LILACS<br>**(n=751)**	(((bariatric surger*) OR (metabolic surger*) OR (stomach stapling) OR (gastroenterostomy) OR (gastroplasty) OR (gastric bypass) OR (mh:(bariatric surgery)))) AND (((preoperative) OR (pre-operative) OR (presurg*) OR (pre-surg*) OR (mh:(preoperative period)) OR (mh:(preoperative care)) OR

	(mh:(preoperative exercise)))) AND (((pharmacological intervention*) OR (pharmacologic action*) OR (drug therapy) OR (pharmacotherapy) OR (glucagon-like peptide-1 receptor agonists) OR (glp-1 receptor agonists) OR (orlistat) OR (anti-obesity agent*) OR (antiobesity agent*) OR (weight loss drugs) OR (mh:(pharmacologic actions)) OR (mh:(drug therapy)) OR (mh:(orlistat)) OR (mh:(glucagon-like peptide-1 receptor agonists)) OR (mh:(anti-obesity agents))) OR ((non-pharmacological intervention*) OR (non-pharmacological action*) OR (diet) OR (exercise) OR (behavioral therap*) OR (behavioral intervention*) OR (lifestyle modification*) OR (combined approach*) OR (gastric balloon*) OR (intragastric balloon*) OR (mh:(diet, reducing)) OR (mh:(preoperative exercise)) OR (mh:(behavior therapy)) OR (mh:(gastric balloon)))) AND instance:"lilacsplus"
BVS **(n = 410)**	(((bariatric surger*) OR (metabolic surger*) OR (stomach stapling) OR (gastroenterostomy) OR (gastroplasty) OR (gastric bypass) OR (mh:(bariatric surgery)))) AND (((preoperative) OR (pre-operative) OR (presurg*) OR (pre-surg*) OR (mh:(preoperative period)) OR (mh:(preoperative care)) OR (mh:(preoperative exercise)))) AND (((pharmacological intervention*) OR (pharmacologic action*) OR (drug therapy) OR (pharmacotherapy) OR (glucagon-like peptide-1 receptor agonists) OR (glp-1 receptor agonists) OR (orlistat) OR (anti-obesity agent*) OR (antiobesity agent*) OR (weight loss drugs) OR (mh:(pharmacologic actions)) OR (mh:(drug therapy)) OR (mh:(orlistat)) OR (mh:(glucagon-like peptide-1 receptor agonists)) OR (mh:(anti-obesity agents))) OR ((non-pharmacological intervention*) OR (non-pharmacological action*) OR (diet) OR (exercise) OR (behavioral therap*) OR (behavioral intervention*) OR (lifestyle modification*) OR (combined approach*) OR (gastric balloon*) OR (intragastric balloon*) OR (mh:(diet, reducing)) OR (mh:(preoperative exercise)) OR (mh:(behavior therapy)) OR (mh:(gastric balloon)))) AND db:("LILACS" OR "IBECS" OR "WPRIM" OR "BINACIS" OR "BDENF" OR "CUMED" OR "BIGG" OR "SES-SP") AND instance:"regional"
Scopus **(n= 20)**	((TITLE-ABS-KEY("bariatric surgery" OR "metabolic surgery") OR TITLE-ABS-KEY("gastric bypass" OR "gastroenterostomy" OR "gastroplasty"))) AND ((TITLE-ABS-KEY("preoperative weight loss" OR "pre-operative preparation" OR "preoperative optimization" OR "presurgical weight loss" OR "pre-surgical preparation"))) AND (((TITLE-ABS-KEY("preoperative pharmacotherapy" OR "preoperative medication") OR TITLE-ABS-KEY("GLP-1 receptor agonists" OR "glucagon-like peptide-1 receptor agonists") OR TITLE-ABS-KEY("preoperative orlistat" OR "preoperative anti-obesity medication"))) OR ((TITLE-ABS-KEY("preoperative diet" OR "pre-surgical diet") OR TITLE-ABS-KEY("preoperative lifestyle intervention" OR "presurgical lifestyle modification") OR TITLE-ABS-KEY("preoperative behavioral therapy" OR "pre-surgical exercise program"))))
Google scholar **(n= 516)**	allintitle: ("bariatric surgery" OR "metabolic surgery" OR "gastric bypass") AND ("preoperative" OR "pre-operative" OR "presurgical")

Table S2. Excluded studies

#	Ref	Year	Reference	Exclusion Reason
**1**	[43]	2024	Rzepa A, Karpińska I, Wierdak M, Pisarska-Adamczyk M, Stefura T, Kawa I, et al. Effect of preoperative intragastric balloon treatment on perioperative and postoperative outcomes after laparoscopic sleeve gastrectomy: A retrospective cohort study. Pol Przegl Chir. 2024;96: 56–62. doi:10.5604/01.3001.0054.2675	Wrong population
**2**	[44]	2019	Fennig U, Snir A, Halifa-Kurzman I, Sela A, Hadas A, Fennig S. Pre-surgical Weight Loss Predicts Post-surgical Weight Loss Trajectories in Adolescents Enrolled in a Bariatric Program. Obes Surg. 2019;29: 1154–1163. doi:10.1007/s11695-018-03649-8	Wrong intervention
**3**	[52]	2022	Mills J, Liebert C, Pratt J, Earley M, Eisenberg D. Complete telehealth for multidisciplinary preoperative workup does not delay time to metabolic and bariatric surgery: a pilot study. Obes Surg. 2022;32: 3605–3610.	Wrong intervention
**4**	[45]	2018	Lemanu DP, Singh PP, Shao RY, Pollock TT, MacCormick AD, Arroll B, et al. Text messaging improves preoperative exercise in patients undergoing bariatric surgery. ANZ J Surg. 2018;88: 733–738. doi:10.1111/ans.14418	Wrong intervention
**5**	[46]	2020	Pouwels S, Sanches EE, Cagiltay E, Severin R, Philips SA. Perioperative Exercise Therapy in Bariatric Surgery: Improving Patient Outcomes. Diabetes Metab Syndr Obes. 2020;Volume 13: 1813–1823. doi:10.2147/DMSO.S215157	Wrong type publication
**6**	[47]	2018	Daniels P, Burns RD, Brusseau TA, Hall MS, Davidson L, Adams TD, et al. Effect of a randomised 12-week resistance training programme on muscular strength, cross-sectional area and muscle quality in women having undergone Roux-en-Y gastric bypass. J Sports Sci. 2018;36: 529–535. doi:10.1080/02640414.2017.1322217	Without comparison
**7**	[48]	2013	Baillot A, Mampuya WM, Comeau E, Méziat-Burdin A, Langlois MF. Feasibility and impacts of supervised exercise training in subjects with obesity awaiting bariatric surgery: A pilot study. Obes Surg. 2013;23: 882–891. doi:10.1007/s11695-013-0875-5	Without comparison
**8**	[49]	2017	Bond DS, Thomas JG, Vithiananthan S, Unick J, Webster J, Roye GD, et al. Intervention-related increases in preoperative physical activity are maintained 6-months after Bariatric surgery: Results from the bari-active trial. Int J Obes. 2017;41: 467–470. doi:10.1038/ijo.2016.237	Multiple reports of the same study
**9**	[53]	2014	Baillot A, Mampuya WM, Dionne IJ, Comeau EM, Méziat-Burdin A, Langlois M. Adding supervised group exercise training to interdisciplinary lifestyle management in subjects awaiting bariatric surgery: a randomized controlled study. 2014;24: 1357. doi:10.1007/s11695-014-1292-0	Multiple reports of the same study
**10**	[54]	2020	Mazowita AE. A nonrandomized trial of a pre-operative physical activity program on bariatric surgery candidates as evaluated by pre- and post-operative physical activity and obesity-related biomarkers. University of Manitoba. 2020. Available: https://mspace.lib.umanitoba.ca/items/a38cd387-9933-4a43-8a4a-02dd91b82322	Multiple reports of the same study
**11**	[55]	2011	NCT01452230. Prebariatric Surgery Physical Activity Program. 2011. Available: https://www.cochranelibrary.com/central/doi/10.1002/central/CN-01533339/full	Protocol
**12**	[50]	2023	Martinez-Huenchullan S, Ehrenfeld-Slater IP, Ehrenfeld-Slater IP. Effects of exercise on physical and metabolic function of candidates before undergoing bariatric surgery. http://isrctn.com/. London, UK; 2023. doi:10.1186/ISRCTN42273422	Protocol
**13**	[56]	2019	NCT03976674. A Preoperative Cognitive Behavioural Therapy Program Based on Self-determination Theory for Bariatric Surgery Candidates. 2019. Available: https://www.cochranelibrary.com/central/doi/10.1002/central/CN-01983212/full	Protocol
**14**	[57]	2009	NCT00830440. A Multicenter Study for Pre-Surgical Weight Loss. 2009. Available: https://www.cochranelibrary.com/central/doi/10.1002/central/CN-02035845/full	Protocol
**15**	[59]	2008	NCT00623792. Study on Impact of Lifestyle Change and Weight Loss Before Bariatric Surgery. 2008. Available:	Protocol

			https://www.cochranelibrary.com/central/doi/10.1002/central/CN-02031284/full	
**16**	[58]	2019	NCT04046367. Prehabilitation in Bariatric Surgery: a Randomized Controlled Clinical Trial. 2019. Available: https://clinicaltrials.gov/study/NCT04046367	Protocol
**17**	[60]	2018	NCT03963986. Impacts of Remote Digital Support on Physical Activity for Patients in Bariatric Surgery (STIMUL). 2018. Available: https://clinicaltrials.gov/study/NCT03963986	Protocol
**18**	[51]	2021	Herrera-Santelices A, Tabach-Apraiz A, Andaur-Cáceres K, Zamunér AR. Effect of physical exercise in bariatric surgery patients: protocol of a randomized controlled clinical trial. Trials. 2021;22: 107. doi:10.1186/s13063-021-05056-4	Protocol
**19**	[61]	2011	NCT03666481. Physical Activity in Bariatric Patients. 2011. Available: https://clinicaltrials.gov/study/NCT03666481	Protocol

Table S3. Funding sources and conflicts of interest of included studies.

Study	Funding Source	Conflicts of Interest
Arman et al. 2021 [42]	No financial support from commercial sources	None declared
Baillot et al. 2016 [43]	Canadian Institutes of Health Research (CIHR Grant No. OPB-131592); FRQ-S scholarships	None declared
Bond et al. 2015 [44]	NIH grant DK083438	One author: BodyMedia research grant (unrelated); Others: none
Creel et al. 2016 [45]	St. Vincent Foundation	None declared
Funderburk et al. 2010 [50]	American Therapeutic Recreation Foundation (ATRF)	Not reported
García-Delgado et al. 2021 [46]	Beca de Investigación Mapfre Guanarteme 2018; Beca Colegio Oficial de Médicos Las Palmas 2019	None declared
Gilbertson et al. 2020 [32]	University of Virginia awards (multiple); American College of Sports Medicine grant	None declared
Hardy et al. 2022 [33]	No financial support received	None declared
Li et al. 2013 [47]	Not reported	Not reported
Marc-Hernández et al. 2019 [51]	Not reported	None declared
Marcon et al. 2017 [48]	Hospital de Clínicas de Porto Alegre (FIPE); CAPES	None declared
Parikh et al. 2012 [49]	2009 SAGES Research Grant Award	Two authors: Allergan Medical Advisory Board (unrelated to study)
Picó-Sirvent et al. 2019 [52]	No external funding	None declared
Picó-Sirvent et al. 2022 [53]	Universidad Miguel Hernández; Fundación MAPFRE; Real Madrid-Universidad Europea	None declared
Still et al. 2007 [54]	Not reported	None reported

NIH: National Institutes of Health; CIHR: Canadian Institutes of Health Research; FRQ-S: Fonds de recherche du Québec-Santé; CAPES: Higher Education Coordination of Improvement; SAGES: Society of American Gastrointestinal and Endoscopic Surgeons

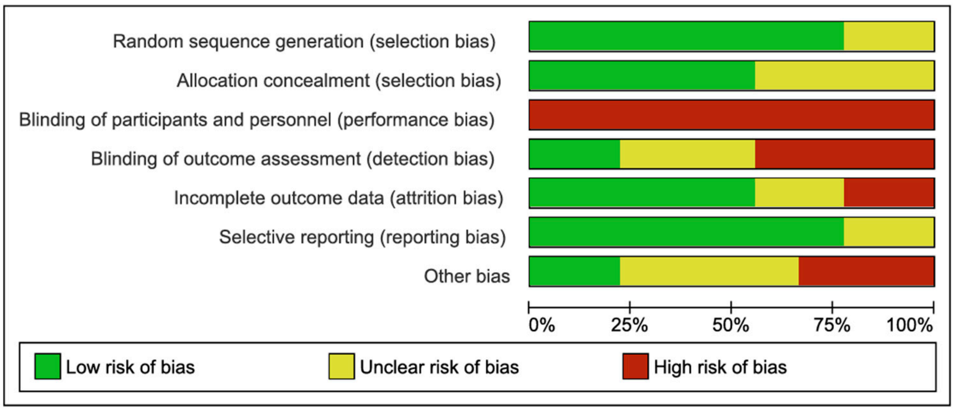

Figure S1. Risk of bias graph (RoB): review authors' judgements about each risk of bias item presented as percentages across all included studies

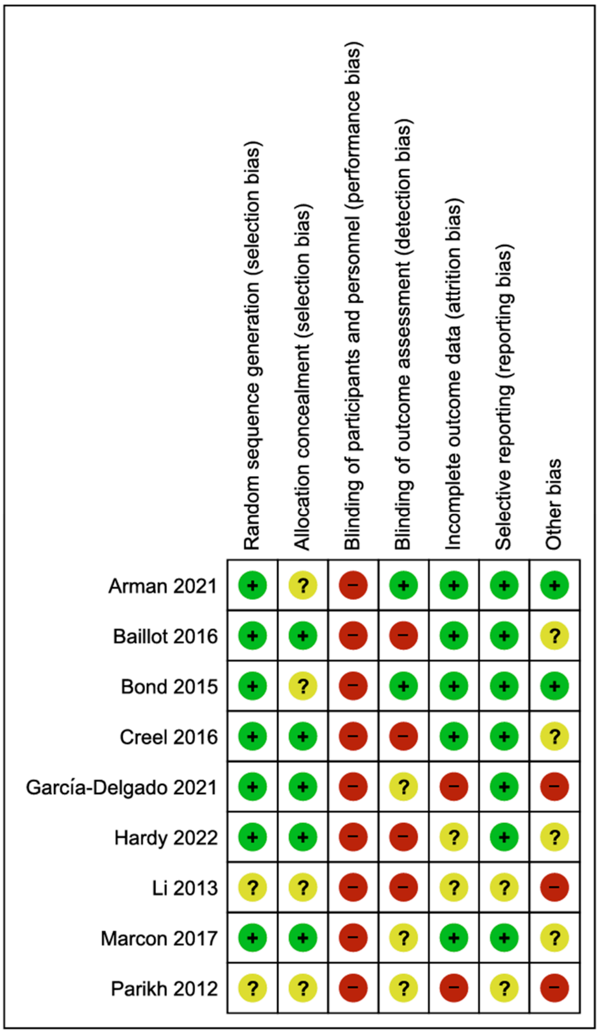

Figure S2. Risk of bias summary (RoB): review authors' judgements about each risk of bias item for each included study.

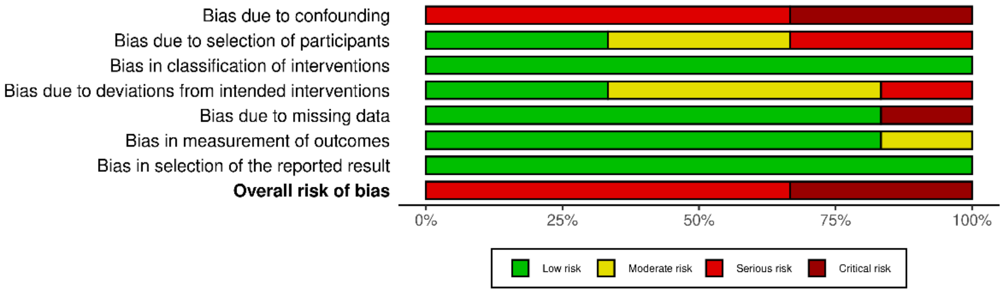

Figure S3. Risk of bias graph (ROBINS-I): review authors' judgements about each risk of bias item presented as percentages across all included studies.

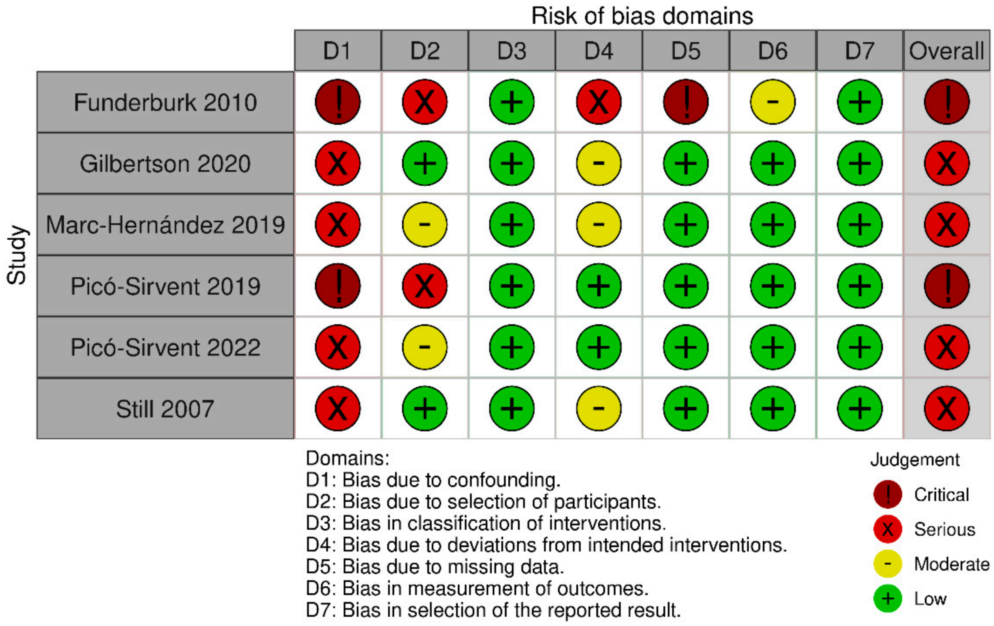

Figure S4. Risk of bias summary (ROBINS-I): review authors' judgements about each risk of bias item for each included study.
